# Species Distribution and Population Connectivity of Deep-Sea Mussels at Hydrocarbon Seeps in the Gulf of Mexico

**DOI:** 10.1371/journal.pone.0118460

**Published:** 2015-04-10

**Authors:** Baptiste Faure, Stephen W. Schaeffer, Charles R. Fisher

**Affiliations:** Department of Biology, The Pennsylvania State University, University Park, Pennsylvania, United States of America; University of Sydney, AUSTRALIA

## Abstract

Hydrocarbon seepage is widespread and patchy in the Gulf of Mexico, and six species of symbiont containing bathymodiolin mussels are found on active seeps over wide and overlapping depth and geographic ranges. We use mitochondrial genes to discriminate among the previously known and a newly discovered species and to assess the connectivity among populations of the same species in the northern Gulf of Mexico (GoM). Our results generally validate the morphologically based distribution of the three previously known GoM species of *Bathymodiolus*, although we found that approximately 10% of the morphologically based identifications were incorrect and this resulted in some inaccuracies with respect to their previously assigned depth and geographical distribution patterns. These data allowed us to confirm that sympatry of two species of *Bathymodiolus* within a single patch of mussels is common. A new species of bathymodiolin, *Bathymodiolus* sp. nov., closely related to *B*. *heckerae* was also discovered. The two species live at the same depths but have not been found in sympatry and both have small effective population sizes. We found evidence for genetic structure within populations of the three species of Bathymodiolinae for which we had samples from multiple sites and suggest limited connectivity for populations at some sites. Despite relatively small sample sizes, genetic diversity indices suggest the largest population sizes for *B*. *childressi* and *Tamu fisheri* and the smallest for *B*. *heckerae* and *B*. sp. nov. among the GoM bathymodiolins. Moreover, we detected an excess of rare variants indicating recent demographic changes and population expansions for the four species of bathymodiolins from the Gulf of Mexico.

## Introduction

Individual species occur in distinct geographic ranges where the members of its populations live, feed, reproduce and die. The current distribution of a species is determined by the suitability of habitats and the colonization and extinction processes over time. Distribution patterns are not permanent and can change as a result of changes in abiotic factors as well as through biotic interactions. Gause’s theory of competitive exclusion [1] suggests that two species with the same ecological niches cannot stably coexist.

When a geographic barrier divides a population, the two separated populations have the potential to evolve into two independent species. New species resulting from allopatric speciation are known as vicariant species. They are often quite similar genetically and occupy very similar niches, but live in geographically separated areas. Secondary contact between such relatively new species is a field of interest for evolutionary ecologists [2]–[4]. Indeed, these closely related species are often phenotypically different where the species occur together (‘sympatry’) but are often indistinguishable where each species occurs alone (‘allopatry’) (reviewed in [5]). When sympatric species are phenotypically similar, postzygotic reproductive isolation can keep co-occurring species distinct [6]. In the case of secondary contact between sibling species, we can distinguish a continuum of contrasting scenarios based on reproductive isolation and/or ecological differentiation. If the species are completely reproductively isolated and sufficiently ecologically differentiated, their interspecific competition will be weak and it is likely that they will coexist in sympatry [3]. On the other hand, if the species are completely reproductively isolated but ecologically identical, interspecific competition will be intense. Principles of competitive exclusion and limiting similarity predict one species will go extinct [7] or diverge through ecological character displacement [8]–[10]. The characters involved in character displacement may be morphological, behavioral, or physiological. Natural selection will favor, in each population, individuals able to use resources or habitats not used by the other species, reducing resource competition and permitting coexistence.

The deep-sea chemosynthetic habitats in the Gulf of Mexico (GoM) provide a natural laboratory to study secondary contact and sympatric habits of species with different levels of differentiation. There are three described species of *Bathymodiolus* in the Gulf of Mexico (*B*. *heckerae*, *B*. *brooksi*, and *B*. *childressi*) [11]. Based primarily on morphology, *B*. *childressi* were reported from 400m to 2200m depth, *B*. *brooksi* from approximately 1080m to 3300m, and *B*. *heckerae* from 2200m to 3300m [12]. Previous phylogenetic analysis of worldwide collections of bathymodiolin mussels revealed that *B*. *brooksi* and *B*. *heckerae* species are closely-related species and belong within the same clade, whereas *B*. *childressi* is in another more divergent clade with other *Bathymodiolus* spp. [13]–[15]. Another deep-sea bathymodiolin, *Tamu fisheri*, is reported from 540m to 700m in the Gulf of Mexico [11], [16] and while most often collected from among vestimentiferan tubeworms is occasionally collected with *Bathymodiolus childressi (CRF pers*. *Obs*.). This species was included in our study because of the risk of misidentification of juveniles, young or broken individuals. Only one species in known in the genus *Tamu* and the phylogenetic position of this unique and divergent lineage remains uncertain [14]–[15].

*Tamu fisheri* harbors thiotrophic symbionts (Species III in [16]), *B*. *childressi* harbor only methanotrophic symbionts, while *Bathymodiolus brooksi* harbor both a methanotroph- and thiotroph-related symbiont and *B*. *heckerae* harbor four co-occurring symbionts (a methanotroph, two phylogenetically distinct thiotrophs and a methylotroph-related phylotype) [17]. *B*. *childressi* are found in a range of physico-chemical environments on the Upper Louisiana Slope, including brine-dominated and petroleum-dominated seep sites, and are exposed to a range of concentrations of methane, oil and hydrogen sulfide [18]. They are also found on the lower Louisiana slope and despite these well differentiated habitats and the large range of depth, there has previously no evidence of significant *B*. *childressi* population subdivision in the GoM [19]. *B*. *brooksi* and *B*. *heckerae* have been collected from a range of environments on the lower Louisiana slope [12], [17], however there is little published data on potential correlations between symbiont complements and environmental conditions.

In this study, we present first an analysis of morphological and genetic diversity for the *Bathymodiolin* mussels from the Gulf of Mexico. We compare these identifications with a barcoding approach, using two mitochondrial genes. We then use additional genetic analyses to test for population structure within species. Finally, we examine the demographic stability of the populations in the Gulf of Mexico and the evolution and interbreeding potential of *Bathymodiolus* species in a larger Atlantic context.

## Material and Methods

### 1. Sample collection

**Fig 1:**
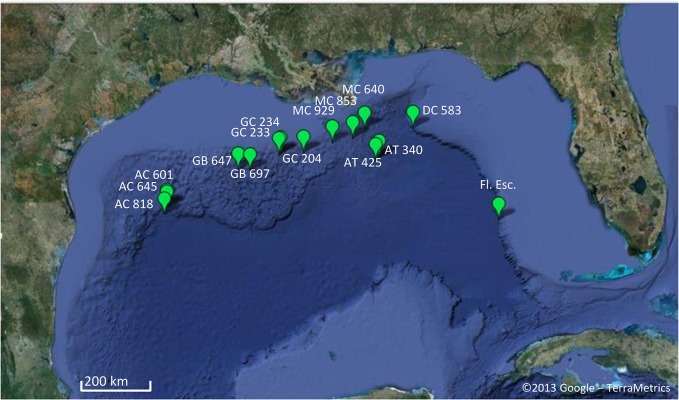
Map of collection sites in the Gulf of Mexico. Sampling effort, depth and coordinates are indicated in Table 1.

Mussels were collected from the deep-sea localities on the Florida Escarpment (Fl.Esc.) and other sites named for the Bureau of Ocean Energy Management Lease Block in which they occur. The lease blocks are designated by a two letter abbreviation for the region (Alaminos canyon, AC; Garden Banks, GB; Green Canyon, GC; Mississippi Canyon, MC; Atwater Valley, AT; Desota Canyon, DC) followed by a three digit number referring to a 3 x 3 nm block. Collections were made using the remotely operated vehicle (ROV) *Jason II* and the *Alvin* and *Johnson Sea Link II* manned submersibles. We extracted DNA and analyzed mussels from 15 discrete sites, ranging from 527 to 3288m and from 84°55’ W to 94°34’ W across the Gulf of Mexico (Fig. 1, Table 1). A total of 238 mussels, collected during 20 dives between October 2002 and August 2009 in the GoM, were included in the analyses. Mussels were collected with either mussel pot collection devices or nets as described in [20]. Upon recovery of the ROV or submersible, the mussels were transferred to chilled seawater, preliminarily identified using morphological criteria, and dissected on board ship. Pieces of tissue (mantle and gills) were frozen (−80°C) or kept in 70% ethanol until nucleic acid extraction using the CTAB+PVP method [21] at The Pennsylvania State University. This work was carried out under a contract from the US Bureau of Ocean Energy Management in the Department of the Interior, the cognizant federal agency charged with overseeing and protecting these communities. All samples were collected in US territorial waters. No permits are required for sampling of these species for scientific purposes in US or international waters.

**Table 1:** Information on mussel samples sorted by depth.

	Site	Latitude	Longitude	Depth (m)	Dive#	Date of collection	*B. childressi*			*B. brooksi*			*B. heckerae*			*B*. nov. sp GoM			*Tamu fisheri*			Accession number	
							Morpho. ID	Genetic ID		Morpho. ID	Genetic ID		Morpho. ID	Genetic ID		Morpho. ID	Genetic ID		Morpho. ID	Genetic ID			
								CO1	ND4		CO1	ND4		CO1	ND4		CO1	ND4		CO1	ND4	CO1	ND4
**Upper slope**																							
	GC234	27°44.1'N	91°13.5'W	527	JSL 4717	17 July. 04	10	10	10	-	-	-	-	-	-	-	-	-	-	-	-	(Bc) KM024170—KM024179	(Bc) KM044648—KM044657
					JSL 4590	05 Sept. 03	-	-	-	-	-	-	-	-	-	-	-	-	5	5	5	(Tf) KM024269—KM024273	(Tf) KM044791—KM044795
	MC929	28°01.1'N	89°43.1'W	636	JSL 3340	08 Oct. 02	15	15	15	-	-	-	-	-	-	-	-	-	-	-	-	(Bc) KM024180—KM024194	(Bc) KM044658—KM044672
	GC233	27°43.4'N	91°16.8'W	651	JSL 4711	09 July 04	10	10	10	-	-	-	-	-	-	-	-	-	-	-	-	(Bc) KM024195—KM024204	(Bc) KM044673—KM044682
	GC204	27°46.0’N	90°32.7’W	870	JSL 3354	17 Oct. 02	15	15	9	-	-	-	-	-	-	-	-	-	-	-	-	(Bc) KM024205—KM024219	(Bc) KM044683—KM044691
	GB647	27°19.8'N	92°25.8'W	1007	J2–280	26 June 07	6	6	0	-	-	-	-	-	-	-	-	-	-	-	-	(Bc) KM024220—KM024225	-
	GB697	27°19.2'N	92°06.7'W	1015	J2–274	17 June 07	4	4	4	-	-	-	-	-	-	-	-	-	-	-	-	(Bc) KM024226—KM024229	(Bc) KM044692—KM044692
	MC853	28°07.6'N	89°08.5'W	1075	AD 4178	14 May 06	3	8	7	9	4	4	-	-	-	-	-	-	-	-	-	(Bc) KM024230—KM024237; (Bb) KM024103—KM024106	(Bc) KM044698—KM044704; (Bb) KM044583—KM044586
**Deeper slope**																							
	MC640	28°21.4'N	88°47.7'W	1414	AD 4182	18 May 06	0	3	2	4	1	1	-	-	-	-	-	-	-	-	-	(Bc) KM024238—KM024240; (Bb) KM024107	(Bc) KM044703—KM044704; (Bb) KM044587
	AT425	27°34.2’N	88°29.6’W	1869	AD 3918	14 Oct. 03	-	-	-	0	3	3	3	0	0	-	-	-	-	-	-	(Bb) KM024108—KM024110	(Bb) KM044588—KM044590
	AC645	26°21.3'N	94°30.1'W	2195	AD 4197	01 June 06	-	-	-	0	3	3	3	0	0	-	-	-	-	-	-	(Bb) KM024134—KM024136	(Bb) KM044607—KM044609
				2197	J2–281	29 June 07	-	-	-	0	3	3	3	0	0	-	-	-	-	-	-	(Bb) KM024137—KM024139	(Bb) KM044610—KM044612
				2200	AD 3923	18 Oct. 03	-	-	-	0	8	7	8	0	0	-	-	-	-	-	-	(Bb) KM024126—KM024133	(Bb) KM044613—KM044619
				2222	AD 3924	19 Oct. 03	27	25	17	15	17	16	-	-	-	-	-	-	-	-	-	(Bc) KM024241—KM024265; (Bb) KM024111—KM024125, KM024140—KM024141	(Bc) KM044705—KM044721; (Bb) KM044591—KM044606
	AT340	27°38.7'N	88°21.9'W	2190	J2–270	10 June 07	-	-	-	4	4	4	-	-	-	-	-	-	-	-	-	(Bb) KM024142—KM024145	(Bb) KM044620 - ‘KM044623
				2216	AD 4180	16 May 06	-	-	-	0	1	1	20	19	19	-	-	-	-	-	-	(Bb) KM024146; (Bh) KM024274—KM024292	(Bb) KM044624; (Bh) KM044724—KM044742
	AC601	26°23.6'N	94°30.9'W	2335	J2–283	07 July 07	3	3	2	-	-	-	-	-	-	-	-	-	-	-	-	(Bc) KM024266—KM024268	(Bc) KM044722—KM044723
	DC583	28°23.1'N	87°23.3'W	2445	J2–454	22 Aug. 09	-	-	-	19	0	0	13	0	0	0	32	32	-	-	-	(BNovSP) KM024071—KM024102	(BNovSp) KM044759—KM044790
	AC818	26°11.1'N	94°34.4'W	2744	J2–282	01 July 07	-	-	-	0	6	6	6	0	0	-	-	-	-	-	-	(Bb) KM024163—KM024168	(Bb) KM044641—KM044646
				2785	AD 4192	27 May 06	-	-	-	16	16	16	2	2	2	-	-	-	-	-	-	(Bb) KM024147—KM024162; (Bh) KM024293—KM024294	(Bb) KM044625—KM044640; (Bh) KM044743—KM044744
	Fl.Esc.	26°01.8'N	84°55.1'W	3288	AD 3915	11 Oct. 03	-	-	-	0	1	1	15	14	14	-	-	-	-	-	-	(Bb) KM024169; (Bh) KM024295—KM024208	(Bb) KM044647; (Bh) KM044745—KM044758
					Total morph. (N = 238)		93	-	-	67	-	-	73	-	-	0	-	-	5	-	-	-	-
					Total CO1 (N = 238)		-	99	-	-	67	-	-	35	-	-	32	-	-	5	-	-	-
					Total ND4 (N = 213)		-	-	76	-	-	65	-	-	35	-	-	32	-	-	5	-	-

Morph, Morphological Sample

JSL, Johnson Sea Link dive number

J2, Jason 2 dive number; AD, Alvin dive number.

(Bc): *Bathymodiolus childressi*

(Bb) *B. brooksi*

(Bh) *B. heckerae*

(BNovSp) *B*. nov. sp. GoM

(Tf) *Tamu fisheri*.

### 2. PCR amplification and sequencing

DNA sequences were obtained for 2 mitochondrial genes: cytochrome c oxidase 1 (CO1) and NADH dehydrogenase subunit 4 (ND4). Fragments of the mitochondrial gene CO1 were amplified using the primers CO1 Bathymodiolus sense/antisense [22], and HCO/LCO [23] with the amplification conditions described in [24]. The ND4 segments were amplified with the sense primers ND46S or ArgBL with the anti-sense primers NAP2H using the conditions described [13].

The PCR products were run on a 1% agarose gel stained with ethidium bromide to check the quantity and the quality of the products and then purified with the ExoSap-It protocol (USB, Affimetrix). Both strands of the purified PCR products for the two mitochondrial genes CO1 and ND4 were directly sequenced (Sanger DNA sequencing, Applied Biosystems 3730XL sequencer at the Huck Institutes, PennState: http://www.huck.psu.edu/facilities).

### 3. Data analyses

Quality of the sequence reads from the two DNA strands were checked from the chromatogram with the Chromas 2.22 computer program (Technelysium Pty. Ltd., Helensvale, Australia). Sequences were aligned with Clustal W [25] in the BioEdit program [26], with manual adjustments to insure that indels were scored consistently among individuals. The distribution of pairwise Kimura’s two-parameter (K2P) distance [27] was plotted for each species. Neighbor-joining trees were constructed using MEGA v4.0 [28] using a K2P distance matrix to infer gene genealogies and 1,000 bootstrap replicates to assess the significance of observed clades [29]. We also included NCBI sequences for *B. thermophilus* (CO1: FJ766893 [30], ND4: AY649808 [31]), *B. azoricus* (CO1: FJ766849 [30], ND4: AF128534 [32]), *B. puteoserpentis* (CO1: FJ766949 [30], ND4: AF128533 [32]) in our phylogenetic trees to further refine relations among taxa. We added CO1 sequences for *B. boomerang* Barbados (DQ513444 [22]), *B. boomerang* Zaire (DQ513442 [22]), *B*. nov. sp. 5°S from the Mid Atlantic Ridge (JQ844817 [33], JQ844789 [33]), and *B*. nov. sp. 9°S MAR (JQ844819 [33], JQ844822 [33]) but ND4 sequences for these species were not available at NCBI.

The number of haplotypes, nucleotide diversity, divergence (average number of nucleotides differences per site between two sequences [34]) and the Tajima’s [35] *D* statistics were estimated using the DnaSP version 4.20.2 software package [36]. Pairwise distances between individuals were used to determine genetic differences among populations based on mtDNA sequences (Φst statistic) with Arlequin [37] and 1,000 permutations of the data were used to evaluate the statistical significance of the differentiation values.

Mitochondrial (CO1) divergence time was estimated as *T = D/2r*, where *D* is the pairwise sequence divergence and *T* is the time of divergence, multiplied by 2 to account for the age of each lineage [38]. To estimate the time of divergence between species, a genus specific CO1 molecular clock was calibrated using the divergence time between *Bathymodiolus thermophilus* from between 13°N and 21°S on the East Pacific Rise and its sister species from 37°S on the East Pacific Rise as in [30]. This particular pair likely split about 5.9 MYA and its CO1 pairwise divergence is 4.62%. Consequently, we estimated a mitochondrial mutation rate of 0.39% per MY. We used this mutation rate (r) to estimate the time of divergence (*T*) between Atlantic species from their pairwise divergence (D).

For the five GoM species of mussels, intraspecific relationships between haplotypes were estimated with the software Network (Network 4.5.1.6, Fluxus Technology Ltd). As our sampling represents only a subset of the natural haplotype diversity, we used the median-joining method [39] to provide the best estimates of the genealogy when internal node haplotype are not sampled [40].

## Results and Discussion

### 1. Morphological and genetic identification

**Fig 2:**
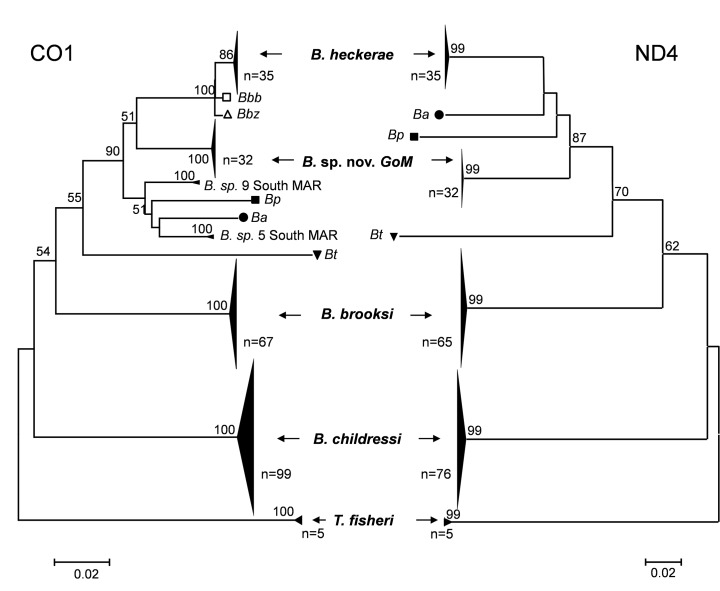
Neighbor-joining trees for mitochondrial loci (CO1 and ND4) using Kimura’s two-parameter method [27]. Bootstrap support above 50% are shown next to the branches (1000 replicates). **CO1**: n = 247 (238 individuals from the Gulf of Mexico, *Bathymodiolus azoricus* from the Mid Atlantic Ridge (Ba: FJ766849), *B. puteoserpentis* from the Mid Atlantic Ridge (Bp: FJ766949), *B. boomerang* from Barbados (Bbb: DQ513444), *B. aff. boomerang* from Zaire (Bbz: DQ513442), *B. thermophilus* from the East Pacific Rise (Bt: FJ766893), *B*. nov.sp 5°S from the Mid Atlantic Ridge (B.sp 5 South MAR: JQ844817, JQ844789), and *B*. nov. sp 9°S MAR (B. sp 9 South MAR: JQ844819, JQ844822). **ND4**: n = 216 (213 individuals from the GoM, *Bathymodiolus azoricus* from the Mid Atlantic Ridge (Ba: AF128534), *B. puteoserpentis* from the Mid Atlantic Ridge (Bp: AF128533), and *B. thermophilus* from the East Pacific Rise (Bt: AY649808)).

The rapid identification of closely related mussels, such as those in the subfamily Bathymodiolinae, at sea is not straight forward, especially for small or incomplete individuals. We used the widely utilized mitochondrial markers CO1 and ND4 to check the species identification of our collections. These markers provide a robust separation of the three previously known GoM *Bathymodiolus* species, *B. childressi*, *B. brooksi*, and *B. heckerae*, as well as numerous other closely related bathymodiolins [13], and the results were 100% consistent between the two markers when both were successfully amplified (we were unable to amplify the ND4 loci for 25 of the 238 individuals). Amplification success and morphological and genetic identifications are presented in Table 1 and the phylogenetic trees are shown in Fig. 2.

Table 2 summarizes the reassignment of the shipboard morphological identifications resulting from the molecular analyses. The discovery of a new monophyletic group of mussels, which we propose is a new species, led to a significant number of misidentifications of *B. brooksi* and *B. heckerae* in that collection. In addition, *B. brooksi* were incorrectly identified on board ship as *B. childressi* 12% of the time and *B. heckerae* were incorrectly identified on board ship as *B. brooksi* 33% of the time. *B. childressi* identified on board ship based on morphological criteria were confirmed with molecular analysis 98% of the time, although as noted above, 12% of the mussels originally identified as *B. brooksi* were later genetically identified as *B. childressi. T. fisheri* were correctly identified based on morphological criteria 100% of the time. Although the original shipboard misidentifications were significant overall, when those associated with *Bathymodiolus* sp. nov. are excluded, about 95% of those remaining were either noted as troublesome in notebooks at sea and/or were associated with small individuals or broken shells.

**Table 2:** Assignment of species after genetic identification (as percentage of total).

	Genetic ID				
Morphological ID	*B. childressi*	*B. brooksi*	*B. heckerae*	B. sp. nov. GoM	*T. fisheri*
From *Bathymodiolus childressi* to…	98 (98)	2 (2)	0	0	0
From *B. brooksi* to…	12 (17)	60 (83)	0	28	0
From *B. heckerae* to…	1 (2)	33 (40)	48 (58)	18	0
From *T. fisheri* to…	0	0	0	0	100

Percentage of assignments after removing individuals of the new species from site DC583 from the calculation are shown in parentheses.

Although the molecular reanalysis of the morphological species identifications changed numerous details with respect to the confirmed sites and relative abundance for the three described species of *Bathymodiolus*, the re-identifications did not result in large range extensions for any species from that previously published [12]. However, the molecular analyses only confirmed *B. heckerae* in three of the original nine collections where the preliminary morphology-based identification indicated they occurred [12]. Our molecular identifications are consistent with previous reports of *B. brooksi* and *B. childressi*, but not *B. heckerae* at the relatively well visited Alaminos Canyon (AC) 645 site [11], [41]-[42] and also confirmed the presence of *B. heckerae* at the nearby but deeper AC818 site. Our analyses support the wide geographic range of this species from east to west across the northern GoM but suggest its upper depth limit is below 2,200m. Our analyses also confirm the widespread occurrence and large depth and geographic range of *B. brooksi* from 1075 to 3288m, and from Alaminos Canyon to the Florida Escarpment. Our analyses slightly extend the already large depth range of the dominant upper slope mussel *B. childressi* from a maximum depth of 2220m in AC645 [42] to 2335m in AC601. Although *B. brooksi* may occur sympatrically with either *B. childressi or B. heckerae*, the latter two species were never found in the same sites. *Tamu fisheri* was only found in sympatry with *B. childressi* (GC234).

A completely unexpected result from our molecular analyses was the discovery of a new species of *Bathymodiolus* (n = 32) at a site in DC583. These mussels are clearly divergent from the 3 other species of *Bathymodiolus* in the GoM, as well as all other *Bathymodiolus* species with published CO1 or ND4 sequences, including those on the Blake Ridge, the North and South Mid-Atlantic Ridge, and the west African Seeps [22], [33], [43–45] (Fig. 2). Two other symbiont-containing species of Bathymodiolinae have also been described in the Gulf of Mexico, *Idas macdonaldi* and *Tamu fisheri* [11], and the new species is genetically distinct from these mussels as well. Phylogenetic trees including closely related mussels confirm the new species belongs in the *Bathymodiolus thermophilus* group as defined by [45] (Fig. 2). Discovery of this new species of *Bathymodiolus* at 2,445m is surprising because this is within the depth and longitudinal range of two other GoM *Bathymodiolus* species (*B. brooksi*: 1075 to 3288m, and *B. heckerae*: 2216 to 3288m depth).

The limited distribution of this new species within the relatively well-sampled GoM and three other widespread species of *Bathymodiolus* suggests that this site is in some way oceanographically isolated from the other sites we have visited. The large size range of animals in our collection (96 to 151 cm in shell length), and the presence of very small mussels on the video record of the site suggests that this population is not the result of a single recent settlement event. *Bathymodiolus* species have a planktonic larval phase and a long larval life period that has the potential for long distance dispersal [46]. Such extensive dispersal potential has been suggested in the amphi-Atlantic distribution of closely related congeners [22], [47]. There is no evidence that closely related species differ dramatically in their dispersal abilities as all the *Bathymodiolus* species studied show evidence of high-rate gene flow and long-range dispersal [48]. Therefore, it is unlikely that this *Bathymodiolus* sp. nov. has limited dispersal ability. We know of no unique current patterns in this area of the gulf that would physically prevent dispersal of *Bathymodiolus* sp. nov. It is possible that *Bathymodiolus* sp. nov. is only able to survive in a very specific habitat that is unsuitable for other bathymodiolins, however we have no data to support this hypothesis and the metabolic versatility of the symbionts of the other GoM *Bathymodiolus* spp. suggest this is unlikely [17]. The occurrence of this species and no other bathymodiolins in this location remains an enigma.

Deep-sea mussels from the GoM are often found in sympatry: *B. childressi* with *T. fisheri* (GC234), *B. childressi* with *B*. brooksi (MC853, MC640, AC645) or *B. brooksi* with *B. heckerae* (AT340, AC818, Fl. Esc.). In sympatry, the principle of competitive exclusion and limiting similarity predict one species will go extinct [7] or diverge through ecological character displacement [8]–[10]. GoM bathymodiolins are clearly in the second situation where sympatric and phenotypically close species diverge through their symbiotic composition [17].Without observation of sympatry between *B. sp*. nov and other *Bathymodiolus* species, we cannot exclude a case of interspecific competition leading to extinction of one species on sites where two species previously occurred. Clearly, additional exploration and oceanographic, evolutionary, and physiological studies are needed to understand the origin and the distribution of this new species of *Bathymodiolus*, and the sympatric occurrence of the others.

### 2. Population structure within mussel species

The power to detect differences with these types of analyses is strongly correlated with sample sizes. Because (as expected) no genetic differentiation was detected between the different sampling dates within a site (at AC645, AT340 and AC818), we combined different collections from the same site for the population structure analysis. Populations with less than three individuals sampled were excluded from these analyses.

**Fig 3:**
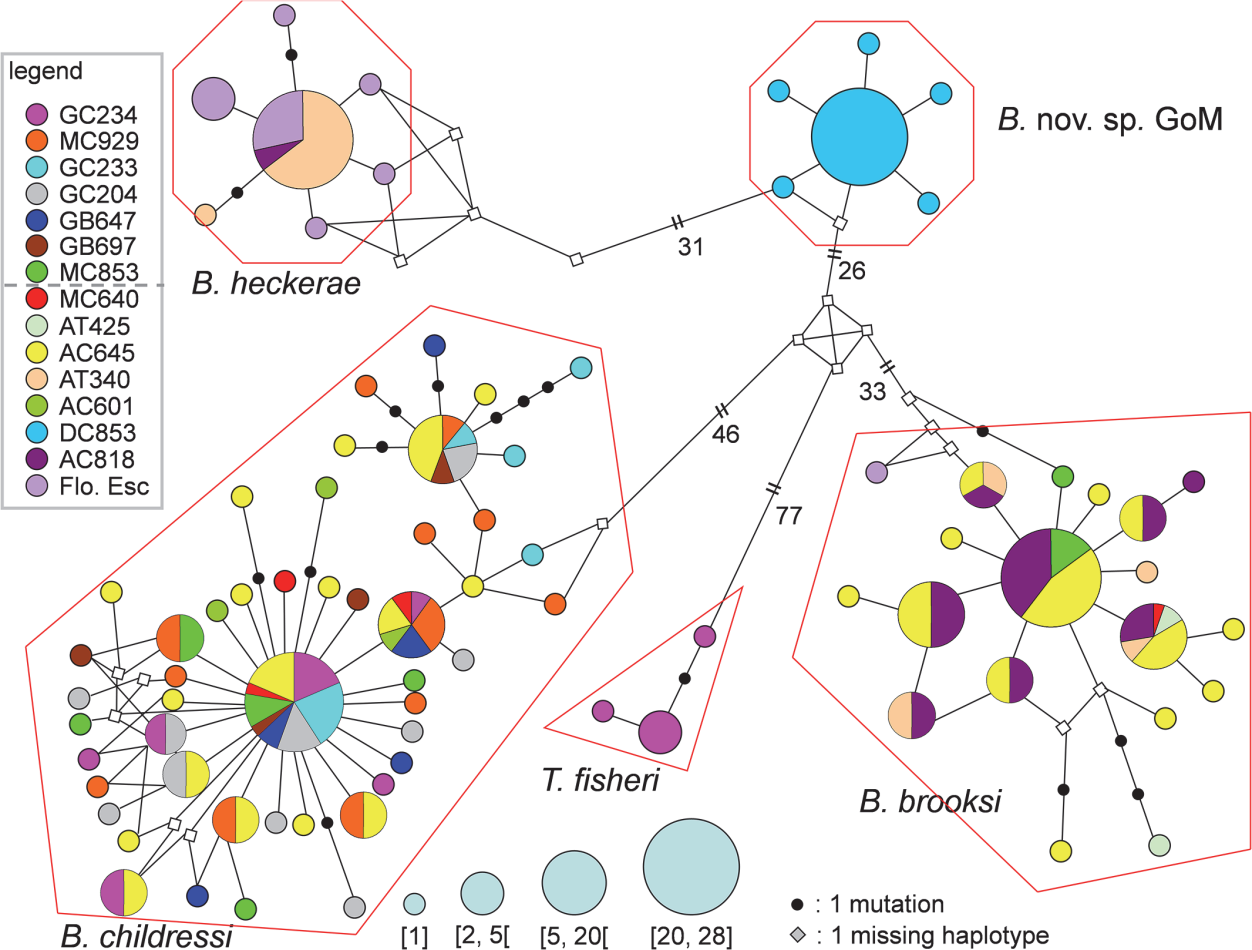
Median-joining network showing genetic structure of the five species of bathymodiolin mussels from the GoM (*Bathymodiolus heckerae*, *B. childressi*, *B. brooksi*, *B*. nov. sp. GoM and *Tamu fisheri*) (CO1 data only). Size of the haplotype circle are proportional to their frequencies (Unique (n = 1); rare (n = 2 to 4), common (n = 5 to 20) and abundant (n = 20 to 28). Black points represent mutations. Diamonds represent missing haplotype. Colours represent locations were the haplotype was found. In the legend, the dashed line represents the virtual limit between “shallow population (less than or about 1000m deep) and deep populations (more than 1400m deep). The number of sequences used for each species and population is indicated on Table 1.

Haplotype networks provide a graphical representation of the genetic structure and relationship among haplotypes within species. The number of haplotypes detected in each species is indicated in Table 3 and the CO1 network of haplotypes for 5 species from the GoM is presented in Fig. 3. In order to maximize the number of individuals for this analysis we used only CO1 (n = 238) (Table 1). Because of the large data set used for this analysis, we simplified the graphical representation of the haplotype network and present only four diameters of haplotype circles (rather than 15 which would otherwise be required): (1) unique haplotypes (n = 1), (2) rare haplotypes (n = 2–5), (3) common haplotypes (n = 5–20), and major haplotypes (n = 20–28).

**Table 3:** Summary statistics for mitochondrial loci polymorphism.

Species	Locus	*N*	*h*	*Hd*	*S*	*π*	Θ_*w*_	Tajima's D
*B. childressi*	CO1	99	48	0.909	45	0.00501	0.01598	−2.24**
	ND4	76	38	0.947	48	0.00465	0.01451	−2.27**
	(CO1+ND4)	76	60	0.988	84	0.00489	0.01488	−2.26**
*B. brooksi*	CO1	67	19	0.829	21	0.00290	0.00807	−1.97*
	ND4	65	25	0.694	30	0.00172	0.00937	−2.62***
	(CO1+ND4)	65	38	0.936	51	0.00226	0.00881	−2.48**
*B. heckerae*	CO1	35	7	0.363	8	0.00094	0.00356	−2.16*
	ND4	35	10	0.494	13	0.00125	0.00466	−2.34**
	(CO1+ND4)	35	13	0.610	21	0.00111	0.00417	−2.49***
B. sp. nov.	CO1	32	6	0.292	5	0.00057	0.00228	−2.01*
	ND4	32	5	0.238	4	0.00037	0.00146	−1.89*
	(CO1+ND4)	32	10	0.490	9	0.00046	0.00183	−2.29**

Locus: CO1 (545bp)

ND4 (678bp)

concatenate C01+ND4: 1123bp)

*N*: number of sequences

*h*: number of haplotypes

*Hd*: haplotype diversity

*S*: Number of segregating sites

*π*: nucleotide diversity

Θ_*w*_: Watterson’s Theta per site from S

Tajima’s D: Tajima’s statistic [35]

Statistical significance

*,P < 0.05

**, P < 0.01

***, P < 0.001

As predicted, every species forms a distinct cluster in the network with no shared haplotypes between species (Fig. 3). The most closely related species (*B. heckerae* and *B*. nov. sp.) are only separated by 33 mutations while the most divergent species (*B. childressi* and *Tamu fisheri*) are separated by at least 126 mutations. *B. heckerae* and *B*. nov. sp. are typical examples of star-like haplotype networks with a single major haplotype (found at 71% and 84% respectively) and a small number of derived haplotypes (n = 7 haplotypes for *B. heckerae*, and n = 6 for *B*. nov. sp.) that are directly derived from the major haplotype.

The *B. childressi* network is more genetically structured than the other species. A total of 48 haplotypes are present with the rare and unique haplotypes articulated around one dominant haplotype (shared by 27 individuals) and two common haplotypes (shared by 9 and 10 indiv.). The majority (81%) of the haplotypes are unique (39 out of a total of 48 haplotypes) and all the unique and rare haplotypes are derived with less than 3 mutations from one of the 3 more common haplotypes. The presence of three major haplotypes may indicate past or intermittent isolation of three sub-populations during its evolutionary history. The haplotypes found at MC853 (in green) are only from (or derived from) the major haplotype. This major haplotype, however, is never found in the well-sampled population MC929 (n = 15, in orange). We detected a significant genetic differentiation between these two populations in the CO1 locus (φ_st_ = 0.106, *p* < 0.05; data not shown in table) suggesting reduced connectivity between these two sites.

Although originally suggested to occur at nine of the study sites [12], *B. heckerae* was confirmed in only three of the study sites (AT340, AC818 and on the Florida Escarpment) and we only collected sufficient sample sizes of individuals from AT340 and the Florida Escarpment for robust population analyses. We detected genetic differentiation in the CO1 locus between AT340 and the Florida Escarpment populations (φ_st_ = 0.04, p<0.01; data not shown). Because of this, we examined the genetic diversity within the populations (Table 4). For both loci, the highest diversity is found for the Florida Escarpment population (for example Fl.Esc. nucleotide diversity (*π* = 0.0018) is almost 5 times higher than AT340 diversity (*π* = 0.0004)). Although variation levels are higher than found in the other GoM populations, this diversity is very low compared to that of other species of *Bathymodiolus* (Atlantic: *π* = 0.0006 to 0.0058 [29], Pacific: up to *π* = 0.003 [56]). *B. heckerae* from AT340 are almost genetically monomorphic for the CO1 locus (only two segregation sites and two haplotypes for 19 individuals, π = 0.00039). This diversity is 4.5 times lower than the diversity detected in samples from the Florida Escarpment. A similar trend is observed with the locus ND4 (π = 0.00147 at the Florida Escarpment and π = 0.00109 at AT340).

**Table 4:** *B. heckerae* site-specific genetic diversity.

Species	Locus	Location	*N*	*h*	*Hd*	*S*	*π*
*B. heckerae*	CO1	Fl.Esc.	14	6	0.681	6	0.00179
	(545bp)	AT340	19	2	0.105	2	0.00039
		AC818	2	1	0	0	0
	ND4	Fl.Esc.	14	5	0.505	7	0.00147
	(678bp)	AT340	19	6	0.468	7	0.00109
		AC818	2	2	1	1	0.00147

*N*: number of individuals

*h*: number of haplotypes

*Hd*: Haplotype diversity

*S*: number of segregating site

π: Average number of nucleotide differences per site between two sequences [34]

The two genes, CO1 and ND4 are in linkage disequilibrium because they are linked on the circular mitochondrial genome. For this reason, we concatenated the two genes and then treated them as a single longer mitochondrial gene for each individual. For this analysis, we removed the individuals for which only one gene was amplified, leaving 213 individuals with concatenated sequences for the population structure analyses (Table 1).

We estimated population differentiation (φ_st_) for *B. childressi* (Table 5), *B. brooksi* (Table 6) and *B. heckerae* (Table 7). Unlike with CO1 alone, no genetic differentiation was detected among the seven *B. childressi* populations nor between the two *B. heckerae* populations in this analysis. This may reflect to lower power due to the reduced sample sizes in key populations. However, two significant differences were detected among *B. brooksi* populations using the concatenated data set: between AT340 and MC853 and between AC818 and MC853. We note that this is consistent with the observation of significant differentiation in the CO1 gene in *B. childressi* from MC853 and suggests limited connectivity between this site and the others sampled in this study. Moreover, and despite several attempts, we were unable to amplify the ND4 locus for any *B. childressi* from the GB647 site. This may result from a mutation on the PCR primer regions and suggest the possibility of reduced connectivity for this population as well.

**Table 5:** *B. childressi* φ_st_ population differentiation, tested by 1023 permutations with the Arlequin software for the concatenated CO1 and ND4 genes.

	Bc_GC234	Bc_MC929	Bc_GC233	Bc_GC204	Bc_GB697	Bc_MC853
	(n = 10)	(n = 15)	(n = 10)	(n = 9)	(n = 4)	(n = 7)
Bc_MC929 (n = 15)	0.01					
Bc_GC233 (n = 10)	−0.03	0.02				
Bc_GC204 (n = 9)	−0.01	0.01	−0.01			
Bc_GB697 (n = 4)	0.01	0.01	0.01	0.01		
Bc_MC853 (n = 7)	0.03	0.03	0.03	0.02	0.03	
Bc_AC645 (n = 17)	0.01	−0.01	0.01	0.01	0.01	0.03

(*: p<0.05)

**Table 6:** *B. brooksi* ɸ_st_ population differentiation, tested by 1023 permutations with the Arlequin software for the concatenated CO1 and ND4 genes.

	Bb_MC853	Bb_AC645	Bb_AT340
	(n = 4)	(n = 29)	(n = 5)
Bb_AC645 (n = 29)	0.07		
Bb_AT340 (n = 5)	0.23*	0.01	
Bb_AC818 (n = 19)	0.15*	0.01	0.01

(*: p<0.05)

**Table 7:** *B. heckerae* ɸ_st_ population differentiation, tested by 1023 permutations with the Arlequin software for the concatenated CO1 and ND4 genes.

	Bh_AT340
	(n = 19)
Bh_Fl.Esc (n = 14)	0.03

**Fig 4:**
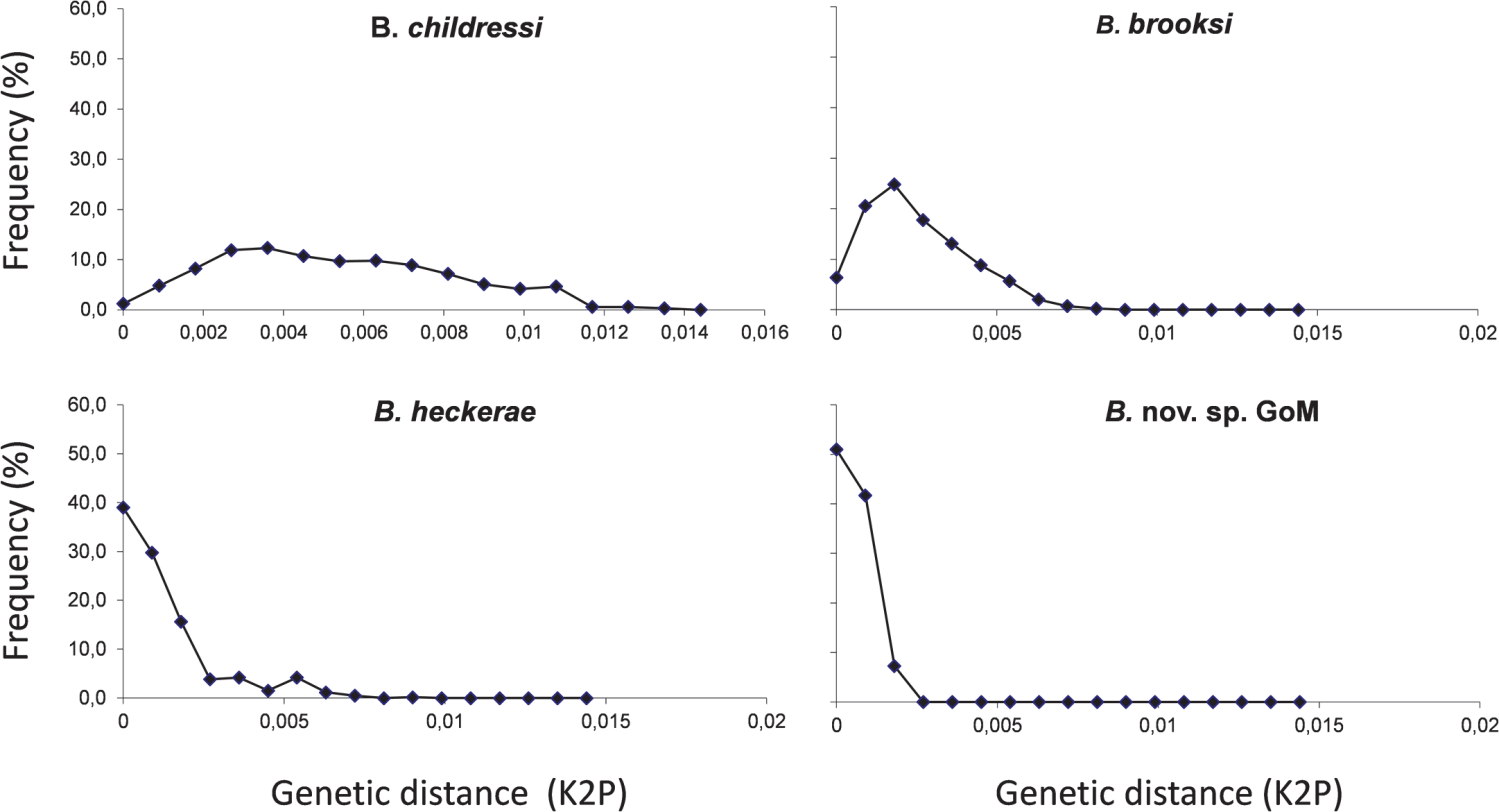
Distribution of pairwise K2P distance-frequency histogram from CO1 and ND4 concatenated data.

Bimodal distributions of pairwise genetic distances can reveal hidden population structure. The distributions of intraspecific distances for the four GoM species are shown in Fig. 4. The highest maximum intraspecific pairwise distance is observed for *B. childressi* (K2P distance = 0.0135) and the smallest maximum distance is observed of *B*. nov. sp. (K2P distance = 0.0018). We never observed a bimodal distribution of the K2P distances which would have been observed if divergent species were analyzed together. Under the assumption of no selection, we reject the hypothesis that any of the bathymodiolin species represent a mixture of deep and shallow water species [49]. The pairwise difference distributions suggest that the populations of *B. childressi* and *B. brooksi* are expanding and that *B. heckerae* and *B*. nov. sp. appear to be at equilibrium [50].

Our preliminary analysis suggests detectable population differentiation in GoM mussel populations against a generally high level of connectivity in the *Bathymodiolus* spp. populations throughout the northern GoM. The data from MC853 and GB647 suggest these populations have limited connectivity with other populations in the GoM and the discovery of a new species limited to a singe site suggests that currently unknown oceanographic or ecological isolating mechanisms may be important in the diversification of these species. Additional studies using larger sample sizes and additional molecular tools such as microsatellites and nuclear markers are needed to better constrain levels of isolation between populations and understand the population biology of the GoM bathymodiolins.

### 3. Low diversity and population expansion in the GoM

As suspected from the haplotype network, levels of genetic diversity are variable between the species. *Bathymodiolus childressi* and *B. brooksi* show a high level of genetic diversity (for example CO1 nucleotide diversity *π* = 0.0050 and 0.0029 respectively) when *B. heckerae and B*. sp. nov GoM nucleotide diversity is almost 3 to 5 times smaller (0.0009 and 0.0006 respectively) (Table 3). This trend (*B. childressi* > *B. brooksi* > *B. heckerae* > B. sp. nov. GoM) is present in both CO1 and ND4.

We used Tajima’s *D* statistic to test the equilibrium conditions in the genetic history of the population. Tajima’s *D* is a difference statistic between two estimates of the neutral mutation parameter (4*N*_e_μ) on a per gene basis, *k* and Θ_w_, where *k* is based on the number of pairwise differences [51] and Θ_w_ is based on the number of segregating sites [52]. A significant difference between the two estimates may indicate the action of selection [35] or demographic changes in the population [53]. The mitochondrial dataset (CO1 and ND4) are considered to be of one locus and were thus analyzed together here. Surprisingly, both concatenated and independent loci have a significantly negative Tajima’s *D* for each species (Table 3), indicating an excess of rare variants. Although an excess of rare mutations is a commonly observed pattern for mtDNA and may be due to segregating slightly deleterious mutations [54], star-like network of haplotypes (Fig. 3), low genetic diversity, and significant negative Tajima’s *D* (Table 3) are strongly suggestive of a non-equilibrium dynamic, which could be interpreted as recent demographic or selective events.

There is no evidence that a selective pressure is acting on the mitochondrial genome. Demographic or geographic population expansion can also result in negative Tajima’s *D*. One can discriminate between these two hypotheses by examining multiple independent loci. Selection is likely to affect just a single locus, but demography is expected to affect all loci in the genome [50]–[56]. This second hypothesis seems more probable and suggests that, despite the relative stability of the cold seep habitats over time (in comparison to the hydrothermal vent habitats of many bathymodiolins), populations are not demographically stable. It could either indicate recent extinction and recolonization of populations (and genetic bottlenecks) or a recent colonization of the GoM by this group. Because of the independent evolution of the four species from the GoM (evidenced by their very dissimilar haplotype networks), a recent or simultaneous colonization seems unlikely. We can only suggest that species and populations in the GoM are not at equilibrium and are subject to a constant flux in population size. This result is similar to those found for animals from deep sea hydrothermal vent habitats [30], [57]. In vent environments, sudden stoppage of hydrothermal activity, plate tectonics and transform faults are all known to promote bottlenecks in populations, recurrence of extinction/recolonization within populations, and allopatric speciation. Changes in seepage appear to also affect cold seep mussel populations, albeit on longer time scale of decades to centuries [58] rather than years to decades as for hydrothermal vent populations [59]–[61]. In both cases the life spans of the mussel species are of the same order as the life span of the habitat [58], [62]. In addition, natural selection may favor some characters for colonization of new habitats (through resistance to greater depths, to certain chemicals, or acquisition of new symbionts). Additional nuclear genetic loci may clarify whether selection or demography are responsible for the population structure observed in the *Bathymodiolus* species in the GoM.

### 4. Evolution and divergence between GoM and Atlantic Bathymodiolinae

In a recent paper, Van Der Heijden et al [33] suggest that the GoM and mid-Atlantic Ridge were not colonized by species coming from the Indian Ridge based on a lack of close relationships between the south mid-Atlantic Ridge and Indian Ocean taxa. Our data document the species richness of *Bathymodiolus* spp. living in GoM (*B. childressi*, *B. brooksi*, *B. heckerae* and *B*. nov. sp. GoM) and also that the GoM and Atlantic species are sister taxa with close phylogenetic relationships (Fig. 2). The phylogenetic relationships need further investigation (with more genes and more species, as, for example in [14] and [15]) to understand the evolutionary scenarios which promoted GoM and Atlantic Bathymodiolus diversity and widespread distribution. Indeed, cold-seep Bathymodiolinae are widespread and are present on both sides of the Atlantic equatorial belt [47]. For example, the *B. childressi* complex is found from the Gulf of Mexico across to the Nigerian Margin and the *B. boomerang* complex is found from Florida escarpment across to the Congo margin. Moreover, Van Der Heijden *et* al [33] suggest there was no evolutionary dispersal barrier for seeps species across the Atlantic Equatorial Belt. Hydrothermal vent *Bathymodiolus* species are also widely distributed: the complex of the closely related and hybridizing species *B. azoricus* and *B. puteoserpentis* extends over 3000km on the Mid-Atlantic Ridge, whereas *B. thermophilus* complex is found over 4600km on the East Pacific Rise (13°N to 38°S) [63]. So far, *B. brooksi* has only been collected in the GoM and, other than the new GoM species detected in this study, is the Bathymodiolinae taxon with the most limited distribution. However, as the Bathymodiolinae often disperse over long distances and new seep sites are still being discovered, we cannot exclude the possibility that other sites, thousands kilometers distant from the documented occurrences of the GoM species, will also harbor *B. brooksi* and/or the new GoM *B*. sp.

We used the mutation rate r = 0.39 [30] to estimate the time of divergence (*T*) between Atlantic species from their pairwise divergence (D) as (T = D / 2r) (Table 8, Table 9) and compared this with the divergence time previously estimated between *B. azoricus* and *B. puteoserpentis* [30]. The results from these two methods are of the same order of magnitude (8.4MYA in this study against 0.37 to 8.6MYA in the literature). Using the same methods we estimate that, *B. heckerae* and *B*. nov. sp. diverged approximately 8 MYA. During such a long divergence time it is unlikely that the new species distribution was restricted to very limited areas in the GoM, suggesting that *Bathymodiolus* sp. nov. will be found elsewhere in the Gulf of Mexico and possibly outside the GoM.

**Table 8:** CO1 divergence between GoM and Atlantic Bathymodiolus species. Divergence is estimated as the average number of nucleotidic differences per site between populations.

	CO1 divergence						
	*B. brooksi*	*B. heckerae*	*B*. nov. sp. *GoM*	*B. azoricus*	*B. puteoserpentis*	*B. boomerang*	*B. sp South* MAR
*B childressi*	0.143	0.144	0.136	0.142	0.145	0.139	0.131
*B. brooksi*		0.121	0.112	0.119	0.125	0.113	0.113
*B. heckerae*			0.064	0.078	0.080	0.011	0.072
*B*. nov. *sp*. GoM				0.078	0.062	0.058	0.059
*B. azoricus*					0.066	0.078	0.056
*B. puteoserpentis*						0.074	0.053
*B. boomerang*							0.064

**Table 9:** Estimated time of divergence between GoM and Atlantic *Bathymodiolus* species (in MY). Time of divergence is estimated using the CO1 mutation rate of 0.39% / MY and divergence values from Table 8.

	CO1 divergence						
	*B. brooksi*	*B. heckerae*	*B*. nov. sp. *GoM*	*B. azoricus*	*B. puteoserpentis*	*B. boomerang*	*B. sp South* MAR
*B childressi*	18.23	18.36	17.34	18.10	18.49	17.72	16.70
*B. brooksi*		15.43	14.28	15.17	15.94	14.41	14.41
*B. heckerae*			8.16	9.94	10.20	1.40	9.18
*B*. nov. *sp*. GoM				9.94	7.90	7.39	7.52
*B. azoricus*					8.41	9.94	7.14
*B. puteoserpentis*						9.43	6.76
*B. boomerang*							8.16

## Conclusion

In a well-studied geographic area such as the GoM, *Bathymodiolus* spp. morphological identifications are generally reliable. They are fast and particularly useful for onboard identifications but present a risk of confusion as soon as new sites are explored and sampled. Genetic barcoding markers work well for the *Bathymodiolus* spp and are appropriate to confirm morphological identifications, especially in cases of juveniles and incomplete individuals and newly discovered sites.

When geographic distance is smaller than the effective dispersal distance, all individuals are potential mating partners. Panmictic populations are generally assumed for species with long-range dispersal capabilities such as *Bathymodiolus* spp. in the GoM [46]. However, although our results are generally in agreement with long range dispersal and considerable connectivity among widely dispersed bathymodilinae populations over a wide depth range, we also find evidence of genetic differentiation within each Bathymodiolus species in the GoM. Even more importantly, a site harboring a completely new species was discovered. Those results are not consistent with complete panmixia of *Bathymodiolus* spp. in the Gulf of Mexico and the as yet unconstrained extrinsic (oceanographic or local chemistry) or intrinsic (selection) factors that drive these patterns need to be investigated.
